# Determination of the optimal working performance matching through theoretical analysis and experimental study for a screw conveyor

**DOI:** 10.1371/journal.pone.0266948

**Published:** 2022-06-16

**Authors:** Xiao Mei, Yukun Xue, Lanlan Zhang

**Affiliations:** 1 Logistics Engineering College, Shanghai Maritime University, Shanghai, China; 2 Shanghai Aviation Service School, Shanghai, China; University of Vigo, SPAIN

## Abstract

An experiment was conducted to investigate the effect of screw speed, feeding head speed, and the nature and type of granular material on the productivity and power consumption of a screw conveyor, and then determine the optimal speed. This experiment contains two parts. Part one was designed with 11 levels of screw speed (100-600r/min, interval 50r/min), three levels of feeding head speed (60,80 and 100r/min), and two kinds of granular materials (fine sand and soybeans). Part two was designed with 11 levels of screw speed (20-140r/min, interval 20r/min), three levels of feeding head speed (300,400 and 500r/min), and two kinds of granular materials (fine sand and soybeans). The results indicated that with the increase of the screw speed, the conveying performance will not always get better; with the rise of the feeding head speed, the conveying performance will get better. Due to the higher bulk density of sand, the productivity of conveying sand is higher for granular materials. Finally, for sand, the result indicates when the screw speed is 400r/min, and the feeding head speed is 120r/min, the conveying performance of sand determined by the productivity and power consumption is the best; for soybean, when the screw speed is 300r/min, and the feeding head speed is 140r/min, the conveying performance is the best.

## Introduction

With the rapid development of containerization and the bulk shipping industry in the world, bulk cargo ship unloading technology has become the core technology of the bulk cargo professional terminal. Therefore, the research of bulk cargo unloading technology and equipment has become the field of material handling technology. Screw conveyor with its high-efficiency conveying capacity, the closed and environmentally friendly conveying condition is widely used in no only port unloading, but also agriculture, food processing, construction, mining, and other industries.

Regarding the conveying performance of the screw conveyor, scholars at home and abroad have conducted many research. Most of them focused on the productivity and power consumption of screw conveyors. Currently, research methods mainly include theoretical analysis, experiment, and simulation. Rademacher [1] developed a theory to describe the behavior of non-cohesive granular material inside a vertical screw conveyor. And the relationships between dimensionless numbers for capacity, power consumption, and efficiency have been derived by using this theory. Orefice et al. [2] performed a set of Discrete Element Method simulations. They found that a smaller shaft always translates into higher throughput and efficiency, and the conveying efficiency was maximized at a filling ratio of around 50%. Owen et al. [3] examined how screw speed, inclination, and filling level influence the performance of a screw conveyor by applying the Discrete Element Method (DEM) to simulate a single-pitch screw conveyor with periodic boundary conditions. According to the simulation results, Gong et al. [4] determined the most suitable for conveying the pitch parameters of silage broken material and screw speed, so that the vertical screw conveyor with high transmission efficiency. Minglani et al. [5] focus on the change in the flow behavior of particles in a screw feeder by changing geometric and operating parameters of screw feeder, using the discrete element method (DEM) simulations software LIGGGHTS. Minglani et al. [6] use Discrete Element Method to quantify the screw feeder performance in terms of average particle speeds (radial, swirl and axial) and contact forces (normal and tangential) by varying screw rotational speed, flight pitch ratio and particle feedrate. Mei et al. [7] used EDEM software and set cycle boundary model to simulate the horizontal single-head screw conveyor for rice and other grain. Tao et al. [8] used ABAQUS software to analyze the dynamic performance of the conveyed material of the screw conveyor. Zheng et al. [9] analyze the particle flow in a screw conveyor with Fluent software. Zhu [10] analyzes the influence of critical parameters such as speed and pitch of the screw structure on the conveying performance of the screw conveyor with the EDEM software.

Due to the inaccuracy of simulation, more and more scholars chose to use experiments for research. Asli-Ardeh et al. [11] conducted an experiment to investigate the effect of screw speed, inclination angle, and variety on the required power and conveying capacity of a screw conveyor. They used three types of paddy grain. Karwat et al. [12] studied the influence of the size of a DEM particle, coefficients of internal and external friction on conveying performance through experiments and simulations, the comparison of the results of the simulations and experiments gave satisfactory results. Li et al. [13] conducted an experiment of pressure distribution and pressure gradient of shield screw conveyor with sandy soil. Owen et al. [14] compared predicted mass flow rates with experimentally measured values. Pezo et al. [15] explored the influences of screw length, particle diameter, the studied geometry variations of screw design, on the mixing performances of the screw conveyor-mixer during material transport through experiment. Tian et al. [16] designed a new screw conveyor with flexible discrete spiral blades and built the power consumption model. Then, its practicability is verified by simulation and experimental testing. Wulantuya et al. [17] studied the feeding methods, running conditions, and flow characteristics of materials to improve the conveying efficiency and working stability of a screw conveyor by theoretical analysis and experiments.

According to the relevant documents, convey capacity and power are two critical performance indicators determined by geometric parameters and the working speed of the screw conveyor. However, there are few comparative experimental studies on the physical properties of granular materials. In previous experimental studies, most scholars only used one kind of granular material, so they cannot compare the difference in conveying performance between different granular materials.

A test-bed of the vertical screw conveyor device was developed based on the conveying characteristics of the vertical screw conveyor. In this paper, we will conduct an experiment with the test-bed of the vertical screw conveyor device to study the influence of vertical screw speed and feeding head speed on productivity and power consumption and determine the optimal speed for the best conveying performance.

## Granular materials and methods

### Testlab details and methods

**Fig 1:**
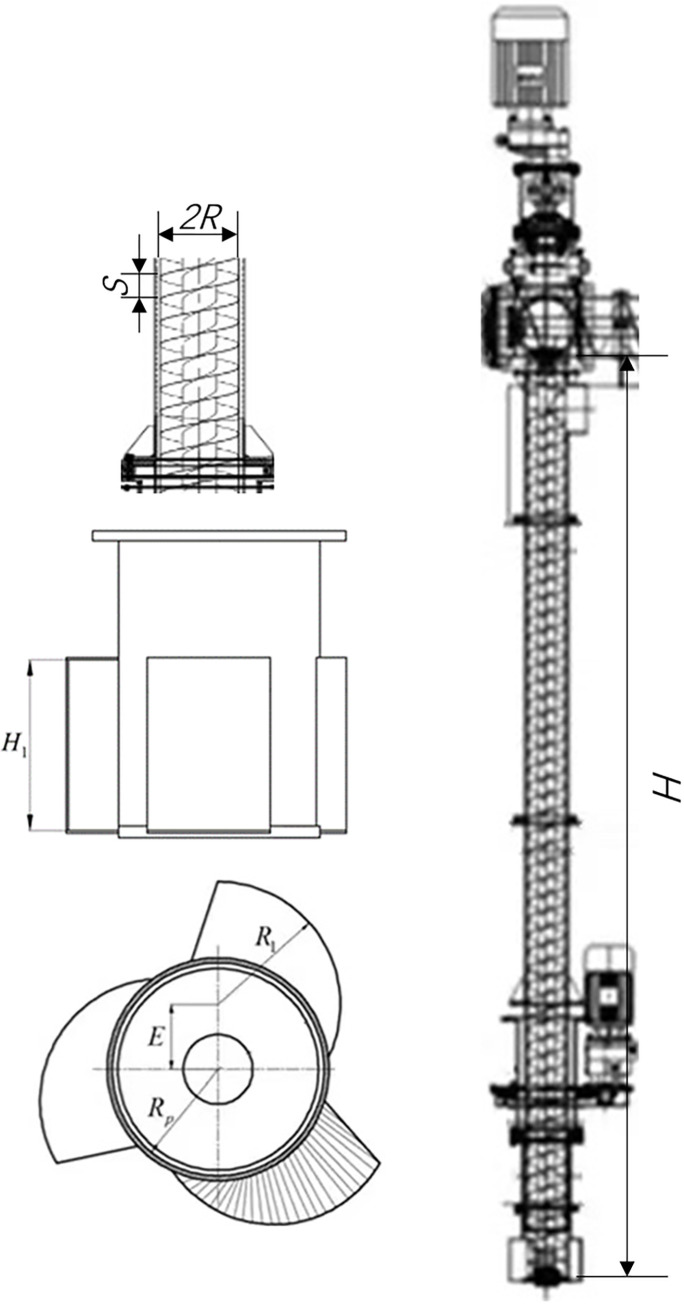
Structure of the vertical screw conveyor.

The structure of the vertical screw conveyor is shown in Fig 1 Two inverters were used to continuously determine the variable transmission of the screw shaft and feeding head. Two rotational speed gauges were used to display the screw rotational speed and the feeding head rotational speed, respectively. A wattmeter was connected to a personal computer for recording and displaying the power data during the tests so that power consumption variations were graphically visible on the monitor during the experiments. Two gravity sensors were used for monitoring productivity.

**Fig 2:**
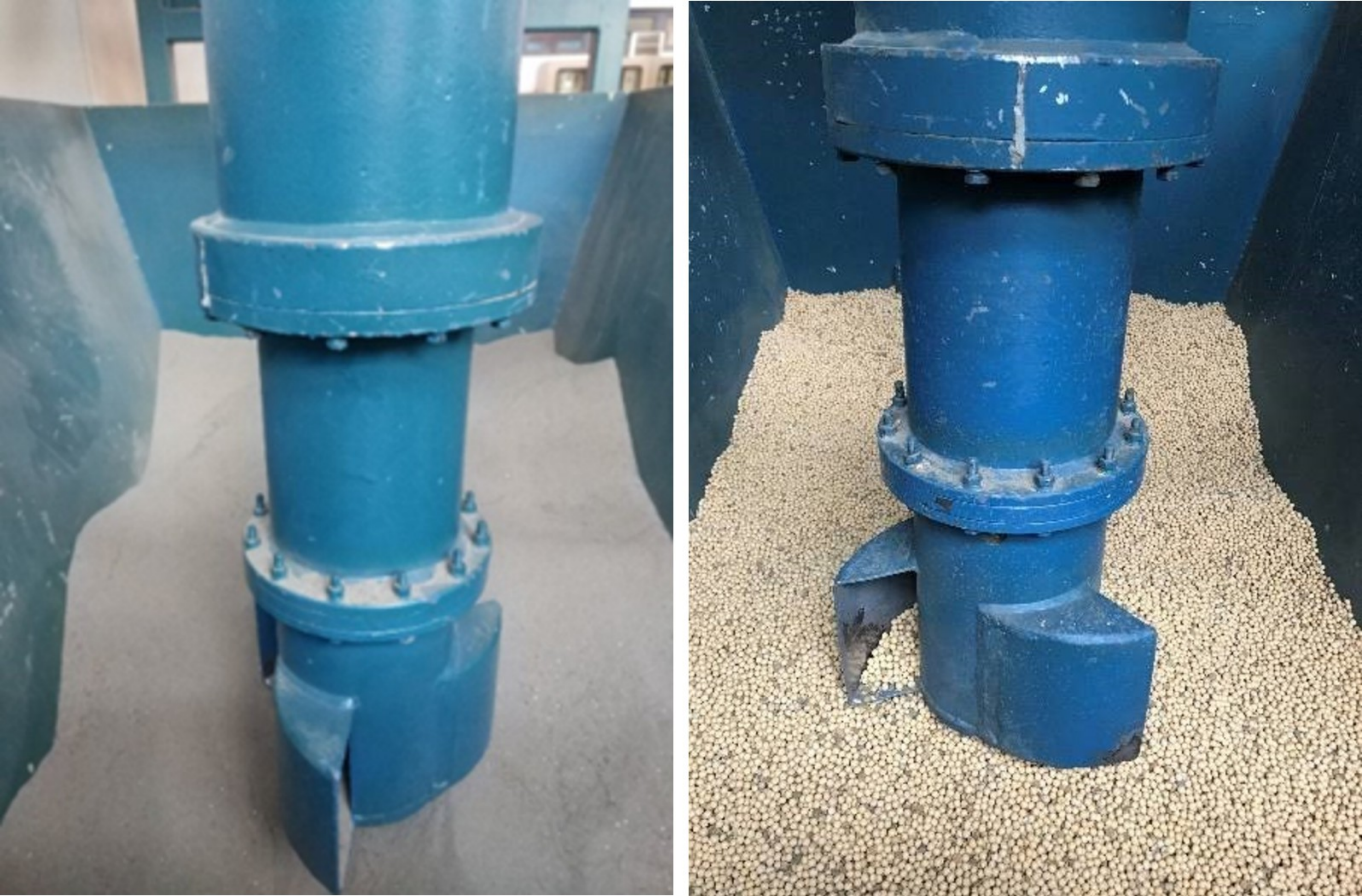
Working mechanism of the vertical screw conveyor. (A) Sand. (B) Soybean.

The granular materials used in this experiment are sand and soybeans, and Table 1 shows the properties of granular materials. Fig 2A and 2B show the working mechanism of the vertical screw conveyor of two kinds of material.

**Table 1:** Granular material characteristic parameters.

Properties	Value	Properties	Value
Filling coefficient of sand *Φ*_1_	0.33	Filling coefficient of soybean *Φ*_2_	0.45
Bulk density of sand ρ_1_	1.485t/m^3^	Bulk density of soybean ρ_2_	0.748 t/m^3^

Specific structural parameters of the vertical screw are as follows: the radius of screw *R* is 140mm, the pitch of screw *S* is 120mm, the vertical conveying length *H* is 3000mm, the height of the feeding head *H*_1_ is 160mm, the arc length of the feed flap on the bottom section of the feed inlet *R*_1_ is 130mm, the radius of the feed pipe *R*_2_ is 77mm, the distance between the axis of the feed wing and the axis of the feed pipe *E* is 27mm, the range of feeding head speed *n*_1_ is 0~140r/min, the range of screw speed *n*_1_ is 0~600r/min, the Rated productivity *Q*_e_ is 15t/h, the rated power *P*_e_ is 5.5kW.

### Mechanical model and theoretical derivation

When the particle is in a stable conveying state, the pressure and friction on the pipe wall, vertical screw shaft, and screw surface, and centrifugal force and gravity, form a dynamic force balance system, which determines the movement characteristics of the particle. When the structure size of the conveyor and the conveyed particle are determined, the characteristic parameter of the particle movement is the particle angular velocity which depend on the feed rate and spiral angular velocity. The relationship between them can be determined by the circumferential equilibrium condition on the particle block per unit circumferential angle on the screw surface.

**Fig 3:**
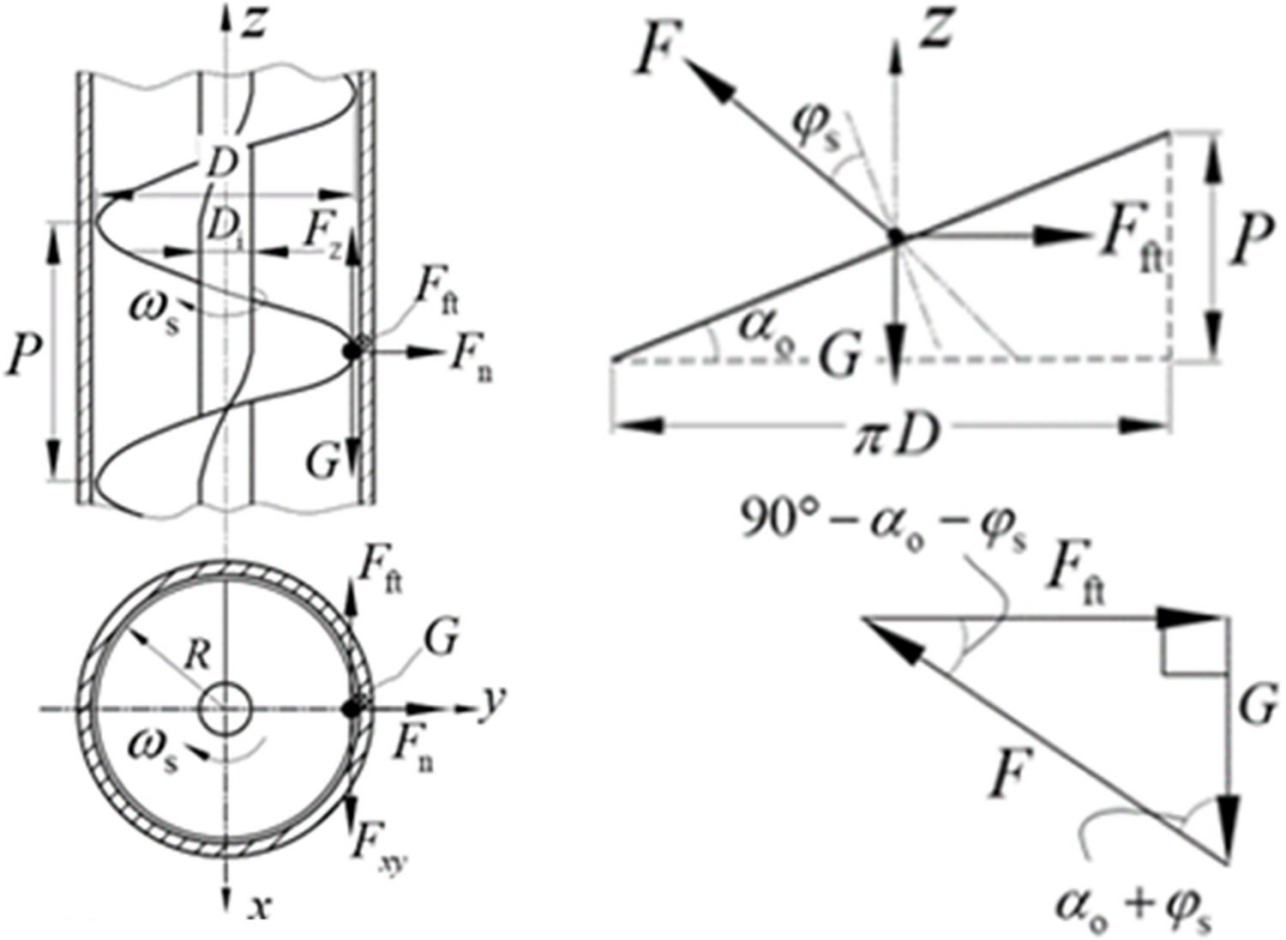
Mechanical analysis of particles. (A) Particle force diagram. (B) Axial direction. (C) Radial direction.


From Fig 3A, we can see that the particle’s gravity G, centrifugal force *F*_*n*_, reaction force *F* of the spiral blade (*F*_*z*_, *F*_*xy*_ are the projections of *F* in the front view and top view) and the friction force *F*_ft_ (*μ*_*t*_ is the friction coefficient) to reach equilibrium. As shown in Fig 3B, in the plane extending along the outer edge of the screw, *G*, *F* and *F*_ft_ form a plane converging force system. As shown in Fig 3C, these forces form a balanced vector triangle. It is easy to know the geometric relationship of the triangle,

$$\tan(\alpha_{o}+\varphi_{s})=F_{\mathrm{ft}}/G.$$ (1)

According to the literature [18], substituting the correlation force into the above formula, we can get

$$\omega_{k}=\sqrt{\frac{g}{\mu_{t}R}\tan(\alpha_{o}+\varphi_{s})},$$ (2)

where *ω*_*k*_ is the critical angular velocity, *g* is acceleration of gravity, *α*_*o*_ is helix angle at the outer edge of the helix, *φ*_*s*_ is friction angle between particle and screw blade. Substitute *ω* = *πn*/30 into the formula (2) to get the critical speed

$$n_{k}=\frac{30}{\pi }\sqrt{\frac{g}{\mu_{t}R}\tan(\alpha_{o}+\varphi_{s})}.$$ (3)

According to the above analysis, the working process of the vertical screw conveyor is as follows,

1. the shaft of the screw structure is driven by a digital frequency motor,
2. then, the screw blade is spinning,
3. finally, the material is entered in the bottom of the screw by the rotation of the feeding head.

When *n*_2_>*n*_*k*_, the material can be conveyed axially.

### Calculation of productivity and power of vertical screw conveyor

The productivity of a vertical screw conveyor refers to the volume or mass of the conveyed granular material per unit time. The unit is generally expressed in t/h, which is an important indicator to measure the conveying efficiency of the vertical screw conveyor. Many factors that influence productivity mainly include geometric parameters and motion parameters, as well as bulk density and filling coefficient of granular material.

With the rotation of the feeding head, the granular material around the wing plate enters the inside of the feeding device from the feeding port. The structural size of the feeding head will affect the sweeping area of the wing plate, and the speed of the feeding head will affect the feeding efficiency. The productivity of the feeding head is

$$Q_{1}=\frac{k_{n}k_{w}H_{1}\rho n_{1}}{0.624\cdot 10^{3}}[{\left(2R_{1}+2E+R_{P}\right)}^{2}-9R_{P}^{2}],$$ (4)

where *Q*_1_ is the productivity of the feeding head, *k*_*n*_ is the number of feeding ports of the feeding head, which can be set as 3, *k*_*w*_ is the influence coefficient of the granular material at the feeding port rotating around the feeding head, which can be set as 1.

Under different influencing factors, the distribution of granular materials within a single pitch is often different. The difference in the free surface of the granular material, on the one hand, reflects the movement of the granular material. The greater the centrifugal force, the more the granular material tends to converge on the outer pipe wall; on the other hand, it can also indicate the filling of the granular material within a single pitch at this time, namely, integrate the free surface in a single pitch plane, and the ratio of the result to the overall distribution area in the pitch is the filling rate of the granular material, which is expressed here. Combined with the axial velocity of the granular material movement, the productivity can be solved, as shown in the following formula,

$$Q_{2}=60\rho \phi \pi R^{2}Sn_{2},$$ (5)

where *Q*_2_ is the productivity of the vertical screw conveyor.

Since the feeding head and the vertical screw are used together, the productivity should also consider the speed and structure of the feeding head and the vertical screw, so take the smaller value of *Q*_1_ and *Q*_2_ as the productivity of the test-bed of vertical screw conveyor

$$Q=\min\{Q_{1},Q_{2}\},$$ (6)

where *Q* is the productivity of the screw conveyor.

According to reference [18], the formula for calculating the shaft power of the vertical screw conveyor is

$$P=\frac{QH}{367\eta_{0}}.$$ (7)

Set the objective function with the goal of maximizing productivity and minimizing power consumption,

$$MaxK=\frac{Q/Q_{e}}{P/P_{e}}.$$ (8)

Set feeding head speed as *n*_1_, and set screw speeding as *n*_2_, so that we can find the best combination of speed to achieve the best conveying performance.

### Experiment method

The experiment parameters are shown in Table 2. Each test starts by pouring 30 kg granular materials into the storage hopper. After the tested screw conveyor was in a stable conveying state, then begin recording experiment data. The test time under single working conditions is 2 min. Productivity data and power consumption data were recorded every 10 seconds for future analysis. The experiment was done twice with two granular materials (sand and soybean), as shown in Fig 3.

**Table 2:** Experiment parameters.

Part	Schem	Speed(r/min)	
		*n*_1_	*n*_2_
1	1	60	100–600, interval 50
	2	80	100–600, interval 50
	3	100	100–600, interval 50
2	4	20–140, interval 20	300
	5	20–140, interval 20	400
	6	20–140, interval 20	500

## Result and discussion

Tables 3–6 respectively show the productivity and power consumption data of sand and soybean in the part 1 of the experiment.

**Table 3:** Sand productivity of scheme 1-3(t/h).

*n*_1_ (r/min)	*n*_2_ (r/min)										
	100	150	200	250	300	350	400	450	500	550	600
60	2.71	4.15	4.77	5.32	5.64	5.88	6.25	6.38	6.54	6.67	6.69
80	2.98	5.27	6.38	7.03	7.58	8.02	8.33	8.67	8.78	8.93	8.95
100	3.19	5.65	6.74	7.87	8.67	9.18	9.61	9.87	10.17	10.61	10.71

**Table 4:** Soybean productivity of scheme 1-3(t/h).

*n*_1_ (r/min)	*n*_2_ (r/min)										
	100	150	200	250	300	350	400	450	500	550	600
60	0.73	1.74	2.21	2.55	2.75	2.84	2.90	2.94	2.98	3.03	3.06
80	1.16	2.03	2.51	2.87	3.16	3.27	3.42	3.54	3.62	3.71	3.77
100	1.30	2.17	2.64	3.04	3.43	3.55	3.77	3.97	4.09	4.20	4.26

**Table 5:** Sand power consumption of scheme 1-3(kW·h).

*n*_1_ (r/min)	*n*_2_ (r/min)										
	100	150	200	250	300	350	400	450	500	550	600
60	0.08	0.09	0.09	0.1	0.1	0.11	0.11	0.11	0.12	0.13	0.13
80	0.09	0.09	0.1	0.1	0.11	0.11	0.11	0.12	0.13	0.14	0.14
100	0.1	0.1	0.11	0.11	0.12	0.12	0.13	0.13	0.14	0.15	0.15

**Table 6:** Soybean power consumption of scheme 1-3(kW·h).

*n*_1_ (r/min)	*n*_2_ (r/min)										
	100	150	200	250	300	350	400	450	500	550	600
60	0.05	0.06	0.06	0.06	0.07	0.07	0.07	0.08	0.08	0.09	0.09
80	0.06	0.06	0.07	0.07	0.07	0.08	0.08	0.08	0.09	0.09	0.09
100	0.07	0.07	0.08	0.08	0.08	0.09	0.09	0.09	0.1	0.1	0.1

**Fig 4:**
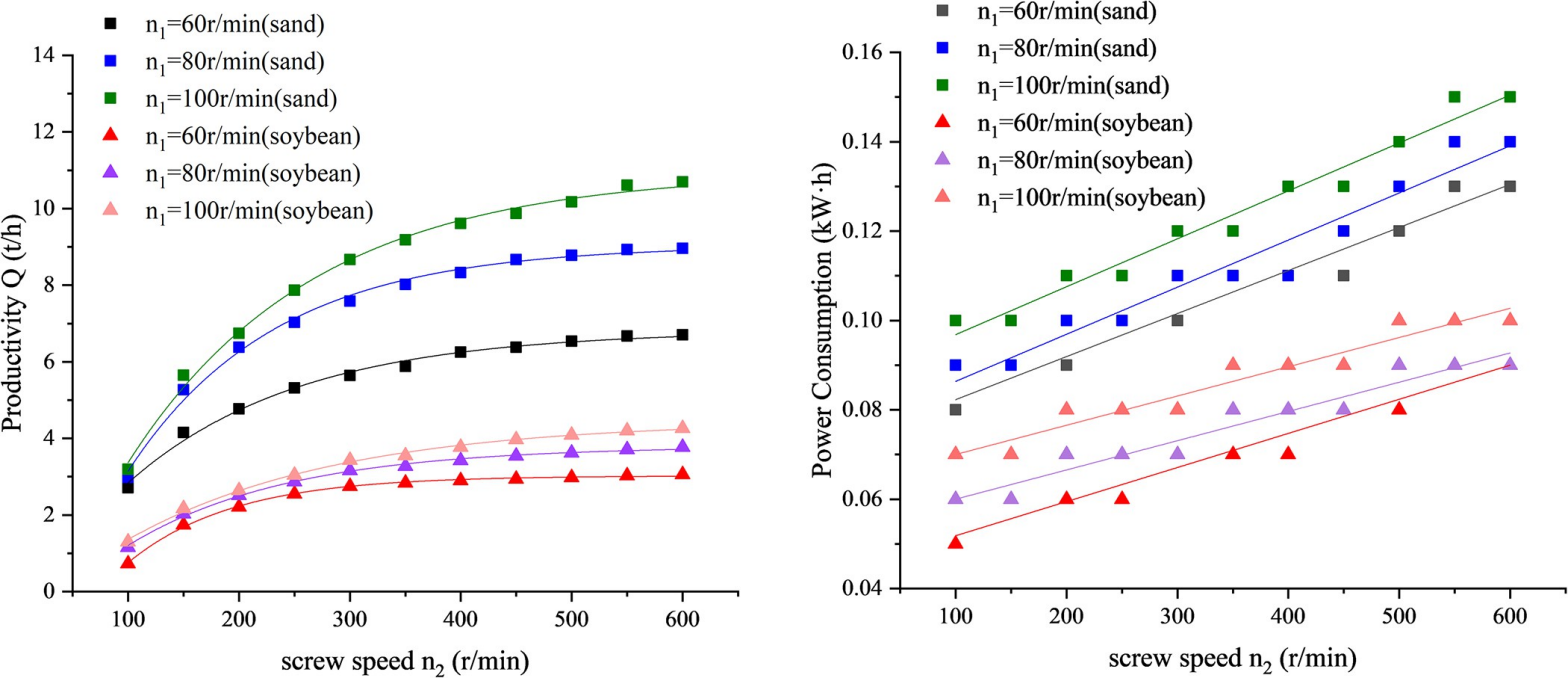
Curve of productivity and power consumption versus screw speed *n*_2_. (A) Productivity. (B) Power consumption.

As shown in Fig 4, by comparing the productivity and power consumption of sand and soybeans under the same experiment parameter, it can be found that the productivity and power consumption of conveying fine sand are about twice that of conveying soybeans, for the reason that the bulk density of sand is twice that of soybeans.

**Fig 5:**
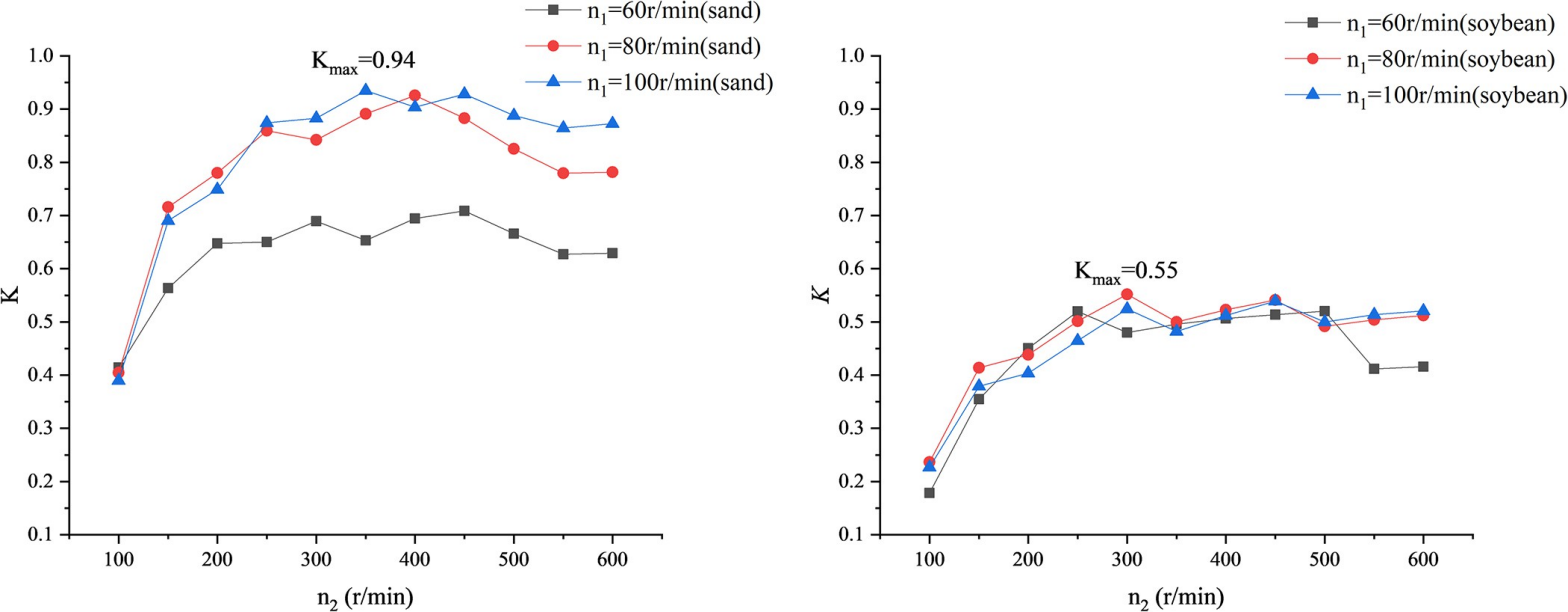
Curve of K versus screw speed *n*_2_. (A) Material: sand. (B) Material: soybean.

To achieve maximum productivity and minimum power consumption. We calculate the dimensionless numbers for productivity and power consumption. The ratio of dimensionless number for productivity to dimensionless number for power consumption is calculated as formula 6. The larger the ratio *K*, the better the conveying effect. Fig 5 is the curve of ratio *K* to screw speed *n*_2_.

As shown in Fig 5A, take sand as an example. When the feeding head speed *n*_1_ is 60, 80, and 100r/min respectively, as the screw speed *n*_2_ increases, the ratio K has a similar trend. When the screw speed increases from 100r/min to 200r/min, The value of K increases approximately linearly, and when the screw speed increases from 300r/min to 500r/min, the value of K has no apparent increasing trend. When the screw speed is greater than 500r/min, the ratio K tends to be flat. In Fig 5B, it can be seen that when soybeans are used as the conveying granular material, the ratio K has the same trend. When the material is sand, the speed of the screw shaft is 350r/min, and the speed of the feeding head is 100r/min, at this time, the value of *K* is the largest, and $\frac{n_{2}}{n_{1}}=3.5$. When the material is soybeans, the speed of the screw shaft is 300r/min, and the speed of the feeding head is 80r/min, at this time, the value of *K* is the largest, and $\frac{n_{2}}{n_{1}}=3.75$. The optimal speed ratio of the two materials is equal.

According to the results of part 1 of the experiment, choose 300, 400, 500r/min as the screw speed *n*_2_ of part 2 of the experiment to explore the relationship between the productivity and the feeding head speed *n*_1_.

Tables 7–10 respectively show productivity and power consumption data of part 2 of the experiment.

**Table 7:** Sand productivity of scheme 4-6(t/h).

*n*_2_ (r/min)	*n*_1_ (r/min)						
	20	40	60	80	100	120	140
300	2.62	4.68	6.25	7.68	8.86	9.91	10.80
400	2.75	5.00	6.86	8.46	9.94	11.00	12.09
500	2.97	5.28	7.11	8.77	10.28	11.40	12.62

**Table 8:** Soybean productivity of scheme 4-6(t/h).

*n*_2_ (r/min)	*n*_1_ (r/min)						
	20	40	60	80	100	120	140
300	0.94	1.86	2.61	3.36	3.78	4.36	4.80
400	1.07	2.03	2.84	3.57	4.20	4.77	5.17
500	1.16	2.26	3.04	3.71	4.39	5.00	5.44

**Table 9:** Sand power consumption of scheme 4-6(kW·h).

*n*_2_ (r/min)	*n*_1_ (r/min)						
	20	40	60	80	100	120	140
300	0.08	0.09	0.1	0.12	0.12	0.14	0.15
400	0.08	0.09	0.11	0.12	0.13	0.14	0.16
500	0.09	0.1	0.12	0.13	0.15	0.16	0.17

**Table 10:** Soybean power consumption of scheme 4-6(kW·h).

*n*_2_ (r/min)	*n*_1_ (r/min)						
	20	40	60	80	100	120	140
300	0.06	0.07	0.07	0.07	0.08	0.09	0.09
400	0.06	0.07	0.07	0.08	0.09	0.09	0.1
500	0.07	0.08	0.08	0.09	0.1	0.1	0.11

**Fig 6:**
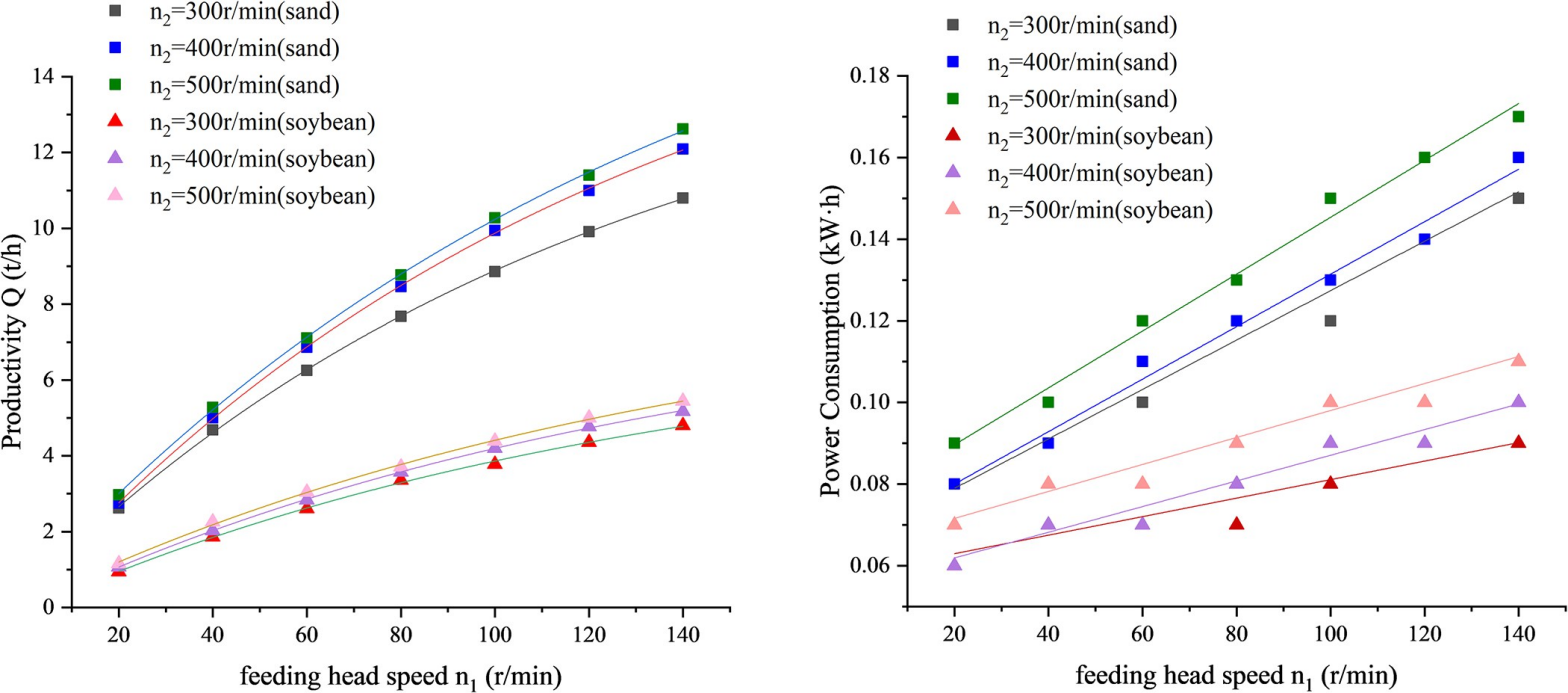
Curve of productivity and power consumption versus screw speed *n*_1_. (A) Productivity. (B) Power consumption.

**Fig 7:**
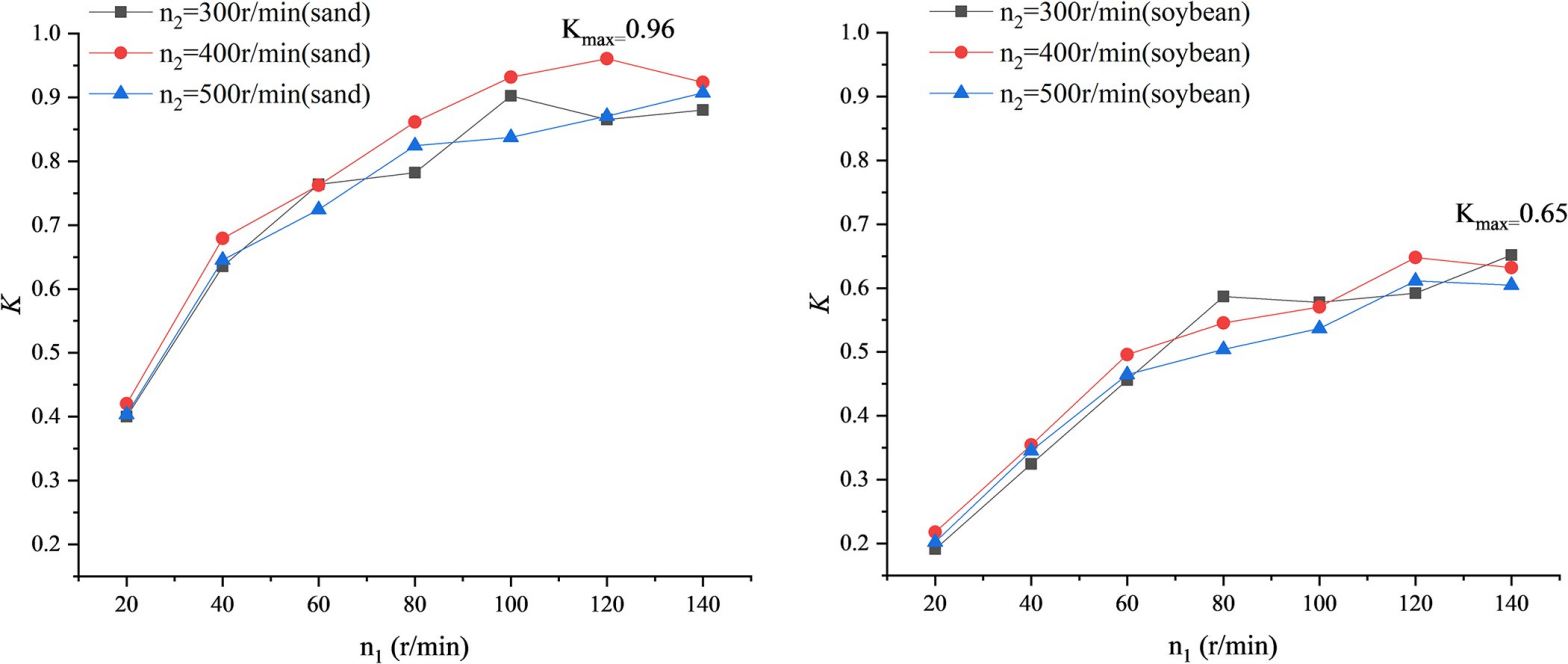
Curve of K versus screw speed *n*_1_. (A) Material: sand. (B) Material: soybean.

As shown in Figs 6 and 7, same as part 1, the productivity and power consumption of conveying fine sand are about twice that of conveying soybeans. After processing the experimental data of productivity and power consumption, Fig 6 is the curve of ratio K to feeding head speed *n*_1_. For both sand and soybean, it can be seen from Fig 7 that when the screw speed is 300, 400, 500r/min, the ratio *K* gradually increases with the increase of the feeding head speed. However, due to the limited range of the feeding head speed of the test-bed of vertical screw conveyor, it is impossible to know what the ratio *K* will change if the speed of the feeding head continues to increase.

To sum up, combined with the analysis of part 1 and part 2 of experiments, for sand, considering the premise of increasing productivity as much as possible and making power consumption smaller, the best screw speed *n*_2_ of the tested vertical screw conveyor can be determined as about 400 r/min, while the best feeding head speed *n*_1_ of is about 120 r/min. And for soybean, the best screw speed *n*_2_ of the tested vertical screw conveyor can be determined as about 300 r/min, while the best feeding head speed *n*_1_ of is about 140 r/min.

When the material is sand, the speed of the screw shaft is 400r/min, and the speed of the feeding head is 120r/min, at this time, the value of *K* is the largest, and $\frac{n_{2}}{n_{1}}=3.3$. When the material is soybeans, the speed of the screw shaft is 400r/min, and the speed of the feeding head is 120r/min, at this time, the value of *K* is the largest, and $\frac{n_{2}}{n_{1}}=3.3$. The optimal speed ratio of the two materials is equal.

**Fig 8:**
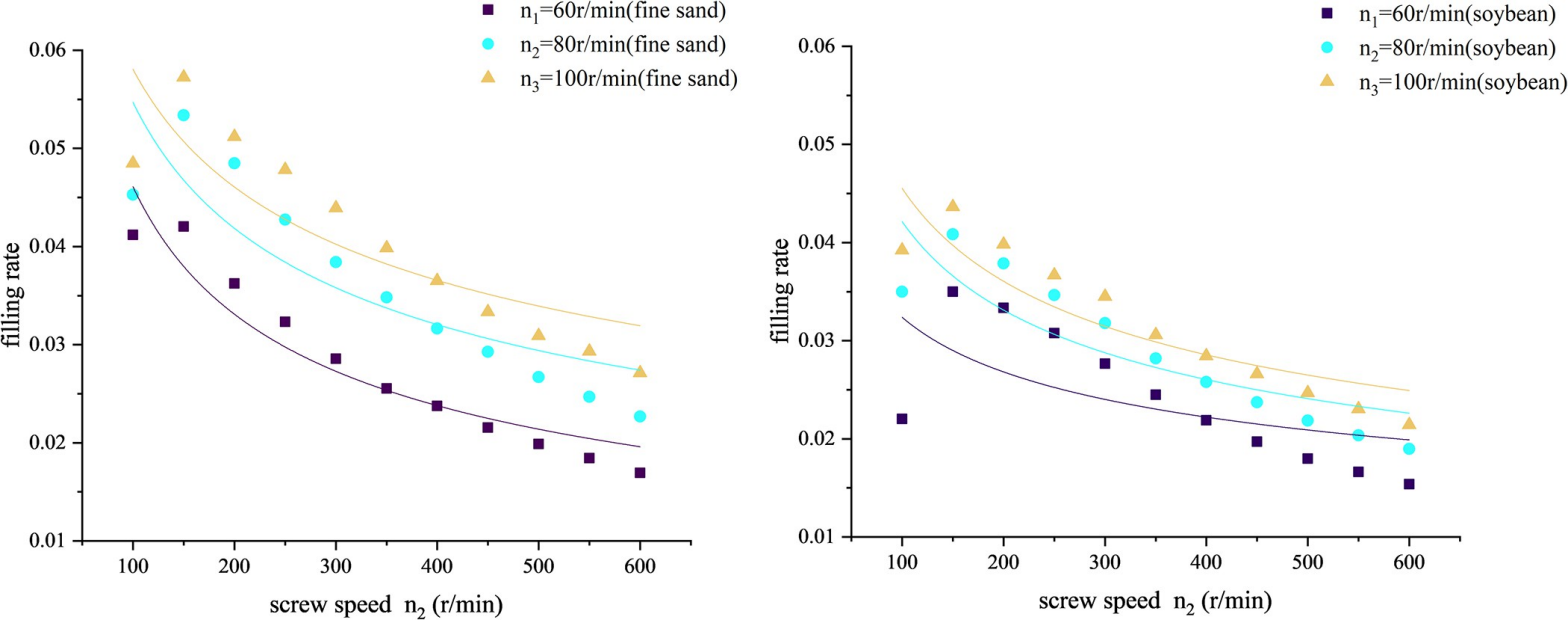
Curve of filling rate versus screw speed *n*_2_. (A) Material: sand. (B) Material: soybean.

As shown in Fig 8, the filling rate decreases with the increase of screw speed *n*_2_. However, the higher the feeding head speed *n*_1_, the higher the filling rate of the screw.

**Fig 9:**
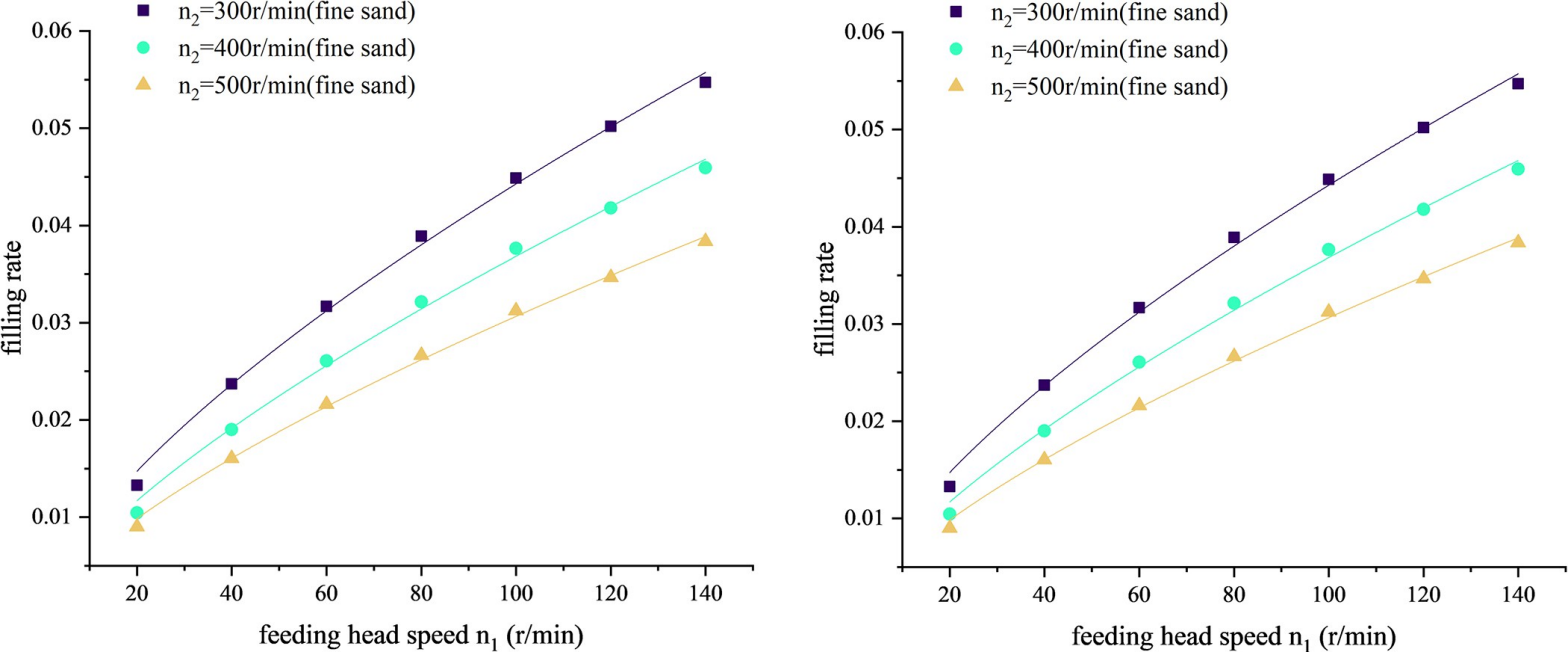
Curve of filling rate versus screw speed *n*_1_. (A) Material: sand. (B) Material: soybean.

As shown in Fig 9, the filling rate increases with the increase of feeding head speed *n*_1_. So, according to formula (4), we can increase the feeding head speed *n*_1_ to increase the filling rate, so that the productivity will increase.

## Conclusions

Through the experiment and related tests and analysis, the following conclusions are obtained:

1. In the existing screw conveyor market, how to determine the optimal performance matching is a crucial problem. In the case of the structure parameters of the screw conveyor cannot be changed, to achieve the best conveying effect, change the screw speed and feeding head speed is the most direct and effective way. Therefore, the method of determining the optimal performance matching in this paper can be used as a reference for other screw conveyors in the market.
2. With the increase of the speed of the screw shaft, the conveying performance will gradually increase and then there will be a downward trend. Therefore, the speed of the screw shaft cannot be blindly increased in the engineering design. The screw conveyor has the best ratio of the screw shaft speed and the feeding head speed, that is, the best speed ratio. The screw conveyor has the best conveying performance under the best speed ratio, and the best speed ratio when transporting sand and soybeans is basically equal, which is 3~4.
3. Comparing the test results of different granular materials, the two granular materials’ test curve has a large difference in productivity and power consumption increase, but the curve trend is similar. The greater the bulk density of the granular material, the higher the productivity, and the power consumption will also increase.
4. The larger the feeding head speed *n*_1_, the larger the filling rate of the screw. So, the productivity can increase by increasing the feeding head speed *n*_1_.
